# Genome-Wide Analysis of the *TCP* Gene Family in Switchgrass (*Panicum virgatum* L.)

**DOI:** 10.1155/2019/8514928

**Published:** 2019-04-09

**Authors:** Yuzhu Huo, Wangdan Xiong, Kunlong Su, Yu Li, Yawen Yang, Chunxiang Fu, Zhenying Wu, Zhen Sun

**Affiliations:** ^1^School of Biology Engineering, Dalian Polytechnic University, Dalian, Liaoning, China; ^2^Key Laboratory of Biofuels, Shandong Provincial Key Laboratory of Energy Genetics, Qingdao Institute of Bioenergy and Bioprocess Technology, Chinese Academy of Sciences, Qingdao 266101, China

## Abstract

The plant-specific transcription factor TCPs play multiple roles in plant growth, development, and stress responses. However, a genome-wide analysis of TCP proteins and their roles in salt stress has not been declared in switchgrass (*Panicum virgatum* L.). In this study, 42 PvTCP genes (*PvTCPs*) were identified from the switchgrass genome and 38 members can be anchored to its chromosomes unevenly. Nine *PvTCPs* were predicted to be *microRNA319* (*miR319*) targets. Furthermore, *PvTCPs* can be divided into three clades according to the phylogeny and conserved domains. Members in the same clade have the similar gene structure and motif localization. Although all *PvTCPs* were expressed in tested tissues, their expression profiles were different under normal condition. The specific expression may indicate their different roles in plant growth and development. In addition, approximately 20 *cis*-acting elements were detected in the promoters of *PvTCPs*, and 40% were related to stress response. Moreover, the expression profiles of *PvTCPs* under salt stress were also analyzed and 29 *PvTCPs* were regulated after NaCl treatment. Taken together, the *PvTCP* gene family was analyzed at a genome-wide level and their possible functions in salt stress, which lay the basis for further functional analysis of *PvTCPs* in switchgrass.

## 1. Introduction

The *TCP* gene family is a class of plant-specific genes encoding proteins with the conserved TCP domain, a 59 amino acid motif that allows DNA binding and protein interaction. The so-called “TCP” is named from four initially identified transcription factors: TEOSINTE BRANCHED1 (TB1) from maize (*Zea mays*), which involved in apical dominance regulation [1, 2]; CYCLOIDEA (CYC) from snapdragon (*Antirrhinum majus*), which controlled floral asymmetry [3]; and the PROLIFERATING CELL FACTORS 1 and 2 (PCF1 and PCF2) from rice (*Oryza sativa*), which are essential for meristematic tissue-specific expression [4]. To date, *TCP* genes have been identified in a number of plant species. For example, there are 24 *TCP* members that were found in Arabidopsis (*Arabidopsis thaliana*) genome, and 28 in rice genome, 30 in tomato (*Solanum lycopersicum*), 21 in medicago (*Medicago truncatula*), 36 in poplar (*Populus trichocarpa*), and 39 in turnips (*Brassica rapa ssp. rapa*) [5–10]. TCP proteins can be divided into two main classes according to their sequences of TCP conserved domain and phylogenetic relationships, which were referred to as class I (also called PCF class or TCP-P class) and class II (also named as TCP-C class) [11, 12]. In angiosperms, class II can be further classified into two clades based on their differences within the TCP domain, which were named as clade CYC/TB1 and clade CIN (CINCINNATA) [5].

TCP proteins play vital roles in plant growth, development, and responses to biotic/abiotic stresses [5, 13]. Class I TCP members were mainly involved in promoting cell proliferation and differentiation by regulating plant hormone signaling, such as gibberellin, auxin, cytokinin, and abscisic acid [13–18]. Class II TCP members were approximately reported to participate in lateral organ development. Furthermore, the origin of clade CYC/TB1 members has occurred later than clade CIN members in angiosperms, and they are primarily involved in shoot branching and apical dominance regulation [5]. TB1 functions as a transcriptional regulator of strong apical dominance and controls the tillering in maize [2]. AtTCP18 (BRANCHED1, BRC1) and AtTCP12 (BRANCHEND2, BRC2) in *Arabidopsis*, two orthologs of maize TB1, are highly expressed in axillary buds and negatively regulate shoot branching [19, 20]. Additionally, *jaw*-*TCPs*, the targets of *miR319* are almost a cluster of CIN members, and *microRNA319*- (*miR319*-) targeted *TCPs* take part in plant cell wall biosynthesis, abiotic stress response, and flowering time regulation in *Arabidopsis* and rice [21–23]. It was also reported that *miR319*-targeted *TCPs* play a role in plant response to salt stress in bentgrass [24, 25]. Besides, some of the *TCP* genes in *Phaseolus vulgaris* which are identified can respond under salt stress [26]. However, the regulation mechanism of TCP transcriptional factors involved in the salt stress has not been elucidated.

Switchgrass (*Panicum virgatum* L.) is a perennial C4 warm-season tall grass used as a bioenergy and animal feedstock for its impressive biomass yield and can confer tolerance to drought, salinity, and poor nutrition [27]. Due to the recent study on high-throughput genome sequencing and assembling, establishment of gene expression atlas, genetic-linkage mapping, and high-efficiency transformation system [28, 29], switchgrass has been developed into the model species as energy grass. The function of numerous genes in switchgrass has been gradually clarified, especially on stress response and development regulation. Until now, WRKY, CCCH, SPL, and ARF gene families had been comprehensively analyzed at the whole-genome level in switchgrass [30–33]. Furthermore, transcriptome microRNAs and long noncoding RNAs exposed to drought stress had been sequenced and analyzed to study the systematic regulatory mechanism of drought response in switchgrass [34–36].

Large amounts of switchgrass will be cultivated on marginal land to avoid competing with food crops for the use of arable fields. Thus, switchgrass regularly faces adverse growth conditions, such as salinity, drought, and extreme temperatures. Analysis has indicated that the *TCP* gene family can respond to salt tolerance, while still little is known about the response of *TCP* genes in switchgrass under a salt stress condition [24, 25]. In this study, a total of 42 *TCP* members were identified in the switchgrass genome. Genome-wide analysis was carried out, including biochemical characterization, phylogenetic analysis, gene structure arrangement, chromosome location, expression profiles of tissue-specific pattern, and responsive pattern under salt stress. Therefore, this work would help us to study the profound functions of the PvTCPs in the future.

## 2. Materials and Methods

### 2.1. Sequence Retrieval and Identification of PvTCPs

The hidden Markov model (HMM) profile of the conserved TCP domain (pfam06507) was retrieved from the Pfam protein family database (http://pfam.sanger.ac.uk/) and used as a query for BLAST searches against the switchgrass genome database in Phytozome v12.0 (*Panicum virgatum* v4.0, DOE-JGI, http://phytozome.jgi.doe.gov/). The candidates were selected for further analysis if the *E* value was less than 1*e* ^−10^. Subsequently, we corrected some errors in annotation of TCP coding sequences on the basis of the switchgrass unitranscript (PviUTs) database (https://switchgrassgenomics.noble.org/) [28]. Finally, all putative PvTCPs were confirmed to be TCP proteins by the Pfam program (http://pfam.xfam.org/), and the peptide length, molecular weight, and isoelectric point parameters of each PvTCP were calculated by the online ExPASy program (https://www.expasy.org/tools/).

### 2.2. Chromosomal Location and Gene Duplication of *PvTCPs*

The lowland switchgrass cultivar, Alamo, is allotetraploid (2*n* = 4*x* = 36) and consists of two highly homologous subgenomes, designated as ChrN and ChrK (*Panicum virgatum* v4.0, DOE-JGI, http://phytozome.jgi.doe.gov/). The chromosomal location of each *PvTCP* was completed using MapChart2.2 based on the physical map in Phytozome v12.0 [37]. Tandem gene duplication was defined as paralogous genes located within 50 kb in tandem and was separated by fewer than five nonhomologous spacer genes [38].

### 2.3. Phylogenetic Analysis of the TCP Proteins

To comprehensively analyze the evolutionary relationships of the TCP proteins in switchgrass, we used putative PvTCPs along with TCP proteins from *Arabidopsis* (model species of dicots) and rice (model species of monocots) to construct a phylogenetic tree. Sequences of the *Arabidopsis* and rice TCP proteins were retrieved from TAIR (https://www.arabidopsis.org/) and rice genome database (http://rice.plantbiology.msu.edu/), respectively. Clustal X1.83 was used to do the multiple alignment of the selected TCPs [39]. The neighbor-joining tree (bootstrap value = 1000) was constructed using MEGA5.0 [40] and then manually improved by the online program EvolView (http://www.evolgenius.info/evolview/).

### 2.4. Gene Structure, Conserved Motif, and *cis*-Acting DNA Element Analysis

The exon/intron structure of *PvTCPs* was determined by comparing the coding sequences and corresponding genomic sequences in the Gene Structure Display Server (GSDS, http://gsds1.cbi.pku.edu.cn/) [41]. Conserved motifs were analyzed using the MEME program (http://meme-suite.org/) [42]. The *cis*-acting DNA element analysis was performed in the promoter sequences (2 kb upstream region) of the *PvTCPs* using the online program PLACE (a database of plant *cis*-acting regulatory DNA elements, https://sogo.dna.affrc.go.jp/). Ka/Ks calculation was analyzed by PAL2NAL [43].

### 2.5. Preparation for Plant Materials

Switchgrass cultivar the lowland Alamo (introduced from the USA and domesticated at Qingdao, China) was used as inbred line for the study. Tissue-cultured seedlings of switchgrass, which can eliminate the interference of genetic background, were subjected to salt stress (about vegetative 3 stage) [44, 45]. During the treatment, 1/2 MS medium supplied with 250 mM NaCl was irrigated [31]. The seedlings irrigated with 1/2 MS medium were regarded as control. Shootings were harvested from three seedlings for each point, and the collection was repeated three times as biological replicates. Samples were frozen immediately in liquid nitrogen and stored at −80°C prior to analysis.

### 2.6. Expression Pattern Analysis of *PvTCPs*

Each of the PvTCPs' transcript sequence was used as a query to blast against the public database of switchgrass (https://switchgrassgenomics.noble.org/). The expression data of spatiotemporal patterns were retrieved, and pretty heatmap was constructed using the online program ImageGP (http://www.ehbio.com/ImageGP/). Total RNA of samples were extracted using the TRIzol method (Invitrogen Life Technologies, USA). The isolated RNA was subsequently treated with RNase-Free DNase I (Roche, http://www.roche.com). The first-strand cDNA was synthesized from 1 *μ*g of total RNA of each sample, using M-MLV reverse transcriptase (TaKaRa, http://www.takarabiomed.com.cn/) according to the protocol. The primers used in this study were showed in Table S1. *PvUBQ* (GenBank accession number: HM209468) was used as the reference gene. qRT-PCR was performed with real-time PCR system (LightCycler 480) using TB Green Premix EX Taq II kit (TaKaRa, Japan) and the methods described in the previous study [32]. Each PCR assay was run in triplicate for three independent biological repeats.

## 3. Results

### 3.1. Identification and Chromosomal Location of *PvTCPs*

**Figure 1:**
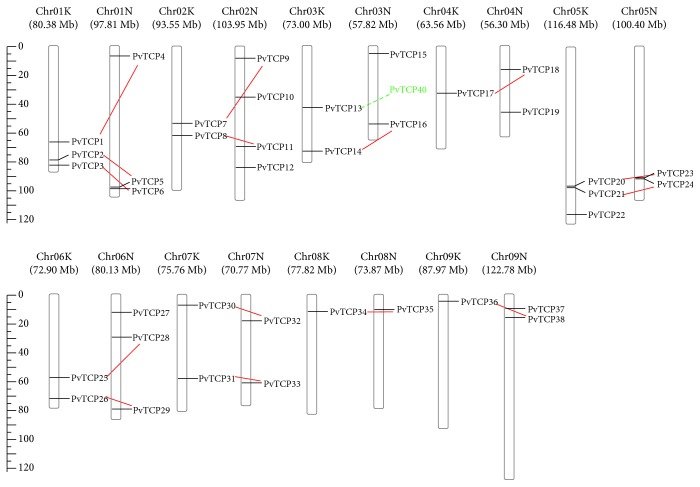
Chromosomal localization of switchgrass *TCP* genes. Chromosomal localization of *PvTCPs* was based on the physical map described in Phytozome v12.0. A total of 38 *PvTCPs* were anchored onto the chromosomes. ChrK and ChrN are two sets of subgenomes of switchgrass (2*n* = 4*x* = 36). The scale on the left represented the physical length of the chromosomes; Mb = million base pair. The red line represented a pair of paralogous *TCP* genes. The green character style represented putative gene pairs.

To identify TCP proteins in switchgrass, the hidden Markov model (HMM) profile of the conserved TCP domain (pfam03634) was used as a blast query to search against the public available switchgrass genome database (Phytozome v12). A total of 42 putative TCP members were identified, which were named as PvTCP1 to PvTCP42 according to their chromosomal location (Figure 1; Table 1). In general, 90.5% (38 out of 42) of *PvTCPs* are anchored onto the chromosomes, while the other four genes are located on an unmapped region. The distribution and density of *PvTCPs* on chromosomes were not uniform (Figure 1). Since switchgrass experienced a whole-genome allotetraploidization (2*n* = 4*x* = 36), the *PvTCPs* exist as paralogous gene pairs in the genome, and the sequence similarity between the gene pairs was larger than 90% (data not shown). 15 pairs of *PvTCPs* are putatively distributed on the ChrN and ChrK, respectively (Figure 1). The numbers for *PvTCPs* on Chr 2, 5, 6, and 7 are two pairs of *PvTCPs*. Chr 1 has three pairs, while Chr 3, 4, 8, and 9 each only has one pair of *PvTCPs* (Figure 1). In addition, according to the results of the specific location of each *PvTCP*, no tandem repeat gene was detected in switchgrass (Figure 1; Table 1).

Biochemical properties of PvTCP members were globally analyzed. Based on the detailed information, lengths of these predicted PvTCP peptides ranged from 94 (PvTCP32) to 450 (PvTCP42) amino acids and molecular weight from 9.70 (PvTCP32) to 46.79 (PvTCP42) KDa (Table 1). The isoelectric point varied from 4.59 (PvTCP32) to 10.42 (PvTCP4) (Table 1).

### 3.2. Phylogenetic Analysis of *TCPs*

**Figure 2:**
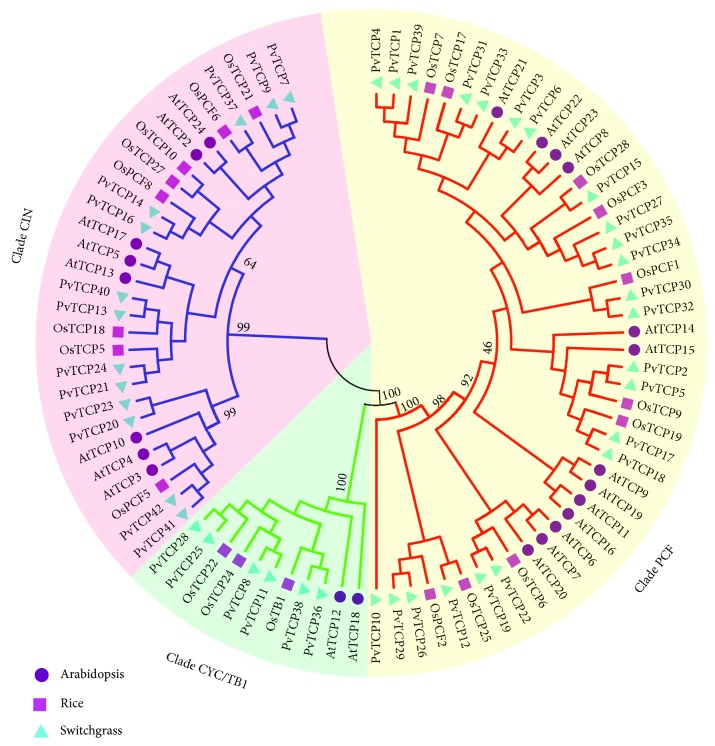
Phylogenetic analysis of TCP proteins in switchgrass, *Arabidopsis*, and rice. An unrooted neighbor-joining (NJ) tree was constructed using MEGA5.0 (bootstrap value = 1,000) after the multiple alignment of peptide sequences. All sequences used in this project were retrieved from the public genome database Phytozome v12.0 (https://phytozome.jgi.doe.gov/pz/portal.html#). The detailed information was listed in Table S2.

**Figure 3:**
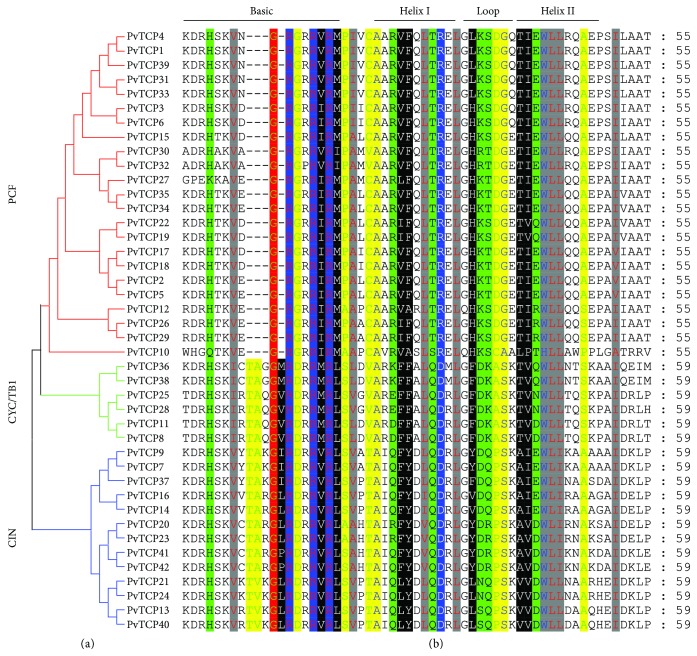
Alignment of the predicted conserved basic helix-loop-helix domain sequence of switchgrass TCP members. Amino acids are expressed in the standard single-letter code. (a) Three clades were classified according to an unrooted NJ tree, which were constructed using PvTCP peptides. (b) Multiple sequence alignment was generated by GenDoc.

In order to comprehensively dissect the function of *PvTCPs*, phylogenetic relationships were firstly analyzed. An unrooted phylogenetic neighbor-joining (NJ) tree was constructed based on the multiple sequence alignments of TCP proteins from switchgrass, Arabidopsis, (model species of dicots) and rice (model species of monocots). Two main classical subfamilies were obviously distinguished according to the NJ tree topology and bootstrap values (higher than 50%), which were referred to as class clades I and II. 23 *PvTCPs* are classified into clade I (PCF), and the rest 19 members are classified into class II (Figure 2; Table S2). The class II group is further divided into clade CIN (13 members) and clade CYC/TB1 (six members) (Figure 2). For the paralogous gene pairs, like *PvTCP1*/*4*, *PvTCP2*/*5*, and *PvTCP3*/*6*, they are all clustered together in the phylogenetic tree, indicating the phylogenetic signature of allotetraploidization (Figure 2). The sequence alignment analysis shows that almost all PvTCP proteins contain the conserved basic helix-loop-helix (bHLH) domain, and the members that belonged to clade I (PCF) have a four amino acid deletions in the bHLH domain compared with class II (CYC/TB1 and CIN) (Figure 3). This result was consistent with the phylogenetic analysis.

*PvTCPs* in both class I and II gathered closely with the counterparts in rice, rather than Arabidopsis, which might imply that *TCP* genes were duplicated after the diversification of dicot and monocot species in angiosperms (Figure 2). Ka/Ks ratios were subsequently calculated between *PvTCPs* and *OsTCPs* (Table 2). The results showed that about 1/3 orthologous genes belonged to purifying selection between the evolution of switchgrass and rice; the other 2/3 orthologous genes belonged to positive selection.

### 3.3. Gene Structure, Conserved Motifs, and Recognition Sites of *miR319*

**Figure 4:**
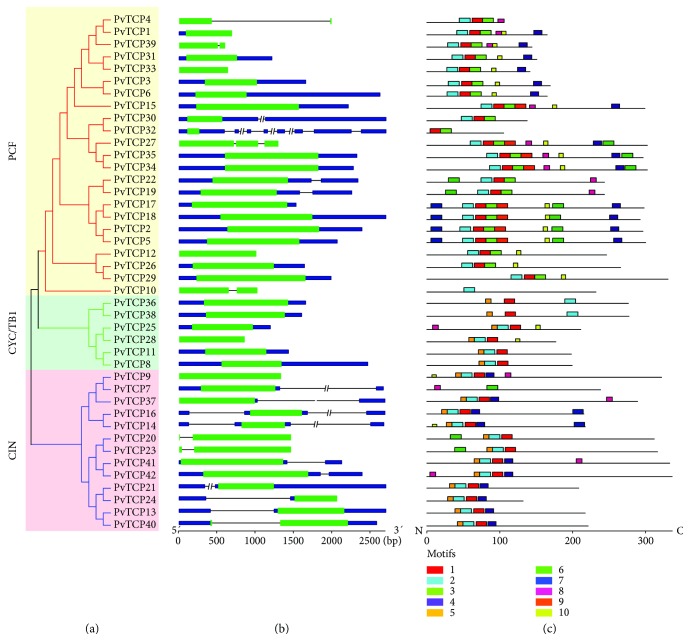
Gene structures and motif locations of switchgrass *TCP* genes. (a) Three clades were classified according to an unrooted NJ tree, which were constructed using PvTCP peptides. (b) Exon/intron arrangements of the *PvTCP* gene. Exons, introns, and untranslated region (UTR) were represented by green boxes, black lines, and blue boxes, respectively. Nucleic acid lengths are indicated by the scale at the bottom; bp = base pair. (c) Schematic representation of conserved motifs in the PvTCP proteins predicted by the MEME program. Each motif is represented by a number in the colored box. The black lines represented the nonconserved sequences. Lengths of motifs for each PvTCP protein were displayed proportionally. aa = amino acid.

To understand the evolution of *PvTCP* gene family, introns in *TCP* genes and conserved motifs of their coding proteins were analyzed (Figure 4). All members in the CYC/TB1 group contain no introns. The intron/exon organization in the PCF clade was relatively conserved, with 14 of 23 members that had no introns, four that had one intron in the coding sequence (CDS) region, one that had two introns in the CDS region, and four that contained one or three introns in the untranslated region (UTR). Introns of *PvTCPs* in clade CIN was not conserved as those in other clades: three contain one intron in the CDS region, nine possessed one or two introns in the UTR region, and only one gene contain no intron (Figure 4). The conserved motifs were also analyzed and ten motifs were identified in PvTCPs using the MEME tool (Figure 4). Motifs 1 and 2 are conserved in PvTCPs except for PvTCP7, PvTCP10, PvTCP32, and PvTCP33. Proteins in the same clade of the phylogenetic tree contain similar motif arrangement. Motif 3 was conserved in all PvTCP proteins of clade PCF except for PvTCP10. Proteins in the other two clades, except for PvTCP7, PvTCP20, and PvTCP23, did not harbor motif 3. This is the same case for motifs 6 and 10. Most PvTCP proteins in clade PCF contain motifs 6 and 10, but not for proteins in clades CYC/TB1 and CIN. Motif 4 was only conserved in clades PCF and CIN, and motif 5 was conserved in clades CYC/TB1 and CIN. Only proteins in clade CIN contain the motif 7. These results implied that TCP transcription factors might take diverse roles in switchgrass due to their structure diversity.

**Figure 5:**
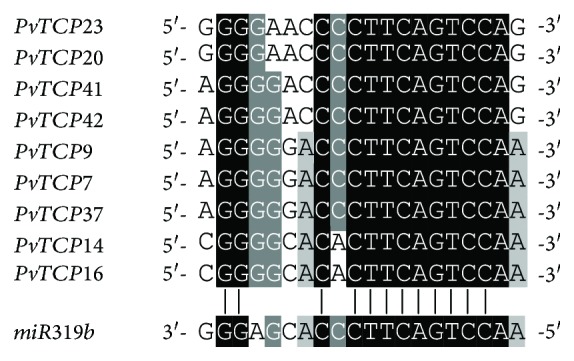
Putative *microRNA319*-targeted binding sites of the *PvTCP*genes. Alignment of complementary pairing bases was generated by GenDoc. Targeted sites were retrieved from the coding sequences of *PvTCP* genes, while mature sequence of *miR319* was rice *miR319b* from miRBase (http://www.mirbase.org/).

It was reported that *TCP* genes can be posttranscriptionally regulated by *miR319* [25]. Similarly, nine *PvTCP* genes contain *miR319* binding sites, which were located in the CDS, and all of these *miR319*-targeted *PvTCPs* were CIN family members (Figure 5).

### 3.4. Tissue Expression Profiles of the *PvTCPs*

**Figure 6:**
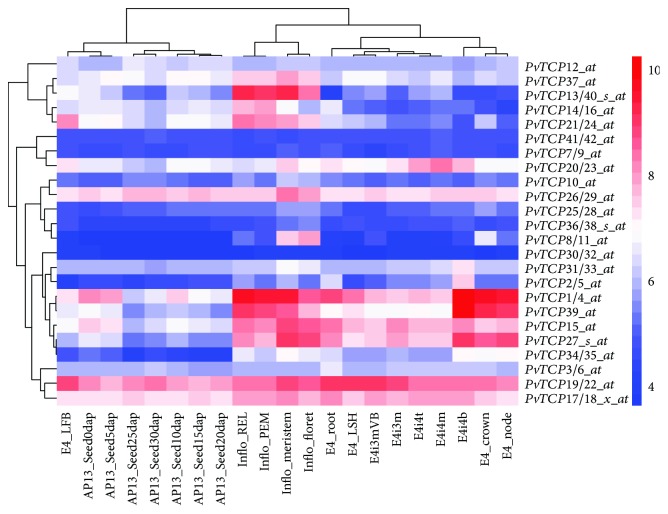
Heatmap of expression profiles of switchgrass *TCP* gene pairs in different tested tissues. The detailed microarray data were obtained from switchgrass gene atlas database (https://switchgrassgenomics.noble.org/). Clustering analysis was carried out using the online program pretty heatmap (http://www.ehbio.com/ImageGP/index.php/). The detailed information was listed in Table S3.

To roundly speculate the function of PvTCP proteins, *cis*-acting DNA elements in the promoter of each *PvTCPs* were retrieved and analyzed (Table S4). The results showed that 18, 15, and 13 elements were, respectively, shared in clades PCF, CYC/TB1, and CIN (Table 3). Obviously, photosynthesis, environmental stress response, and phytohormone regulation were the three major aspects in which TCP proteins were involved. In order to deeply analyze the tissue expression profiles of the *PvTCP* family, microarray data was obtained from the public database. As expected, both *PvTCPs* of the gene pair share one probe (Table S3). All *PvTCPs* were expressed in the examined tissues (leaf, node, internode, root, flower, and seed) (Figure 6). Part of the genes in the same clade exhibited similar expression mode. For example, members in clade CIN (*PvTCP37*, *PvTCP13*/*40*, *PvTCP14*/*16*, and *PvTCP21*/*24*) predominantly expressed in flowers, which might take roles in pollen development. Genes in clade PCF, like *PvTCP1*/*4*, *PvTCP39*, *PvTCP15*, and *PvTCP27*, represented a high expression level in flowers, node, and seed of E4 stage. Besides, *PvTCP26*/*29*, *PvTCP19/22*, and *PvTCP17*/*18* displayed high expression levels in all tested tissues, while *PvTCP10*, *PvTCP25*/*28*, *PvTCP36*/*38*, *PvTCP30*/*32*, *PvTCP7*/*9*, and *PvTCP41*/*42* were expressed relatively low in all tested tissues.

### 3.5. Gene Expression Response of *PvTCPs* under Salinity Condition

**Figure 7:**
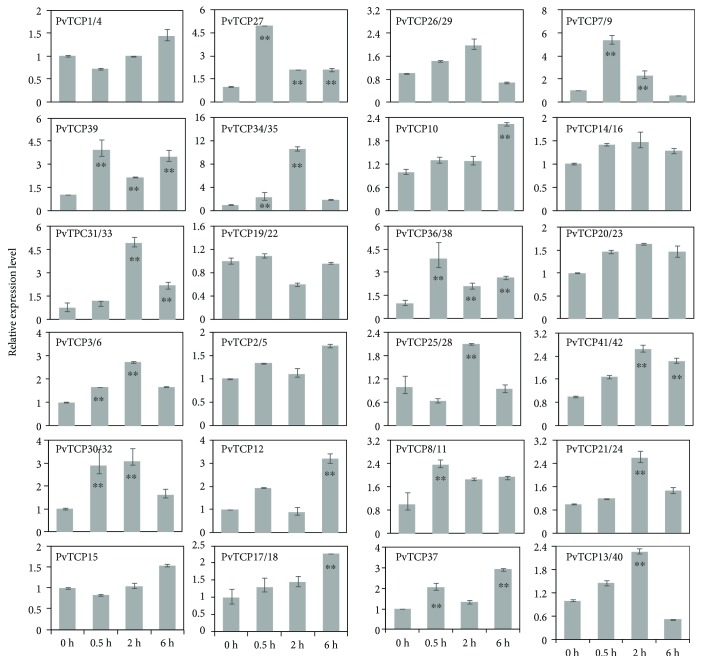
The expression of *PvTCP* genes in response to treatment with 250 mM NaCl for 0.5, 2, and 6 hours in seedlings. Control plants were collected before the treatment by NaCl solution. Error bars represented variability of three independent replicates. Statistically significant differences were assessed using Student's *t*-tests (^∗∗^represented *p* ≤ 0.01).

Based on the statistical results from *cis*-acting DNA elements, about 40% were showed to respond to environmental stress, especially to salinity (Table 3). To explore the expression profiles of *PvTCP*s under salinity condition, 42 *PvTCPs* were analyzed by qRT-PCR (Figure 7). 29 *PvTCP*s were regulated under salinity condition, and the other 13 *PvTCPs* were not statistically significant after 6 h salt stress (Figure 7). 14 out of 23 (about 60.8%) *PvTCPs* in clade PCF were upregulated during the 6 h salinity treatment. Of these genes, *PvTCP27* and *PvTCP39* were showed upregulated in all three treatment points. *PvTCP3*/*6*, *PvTCP30*/*32*, and *PvTCP34*/*35* were upregulated at 0.5 h and exposed to salt stress for 2 h, and recovered to the normal expression level at 6 h treatment point. *PvTCP10*, *PvTCP12*, and *PvTCP17*/*18* were upregulated at 6 h treatment point. *PvTCP31*/*33* was induced after 2 h treatment. All *PvTCPs* in clade CYC/TB1 were upregulated. Similarly, nine out of 13 *PvTCP* genes in clade CIN were upregulated after 6 h treatment. These results showed that a large number of *PvTCPs* were response to salt stress and displayed different expression profiles when exposed to salinity condition.

## 4. Discussion

The *TCP* gene family is a cluster of plant-specific transcription factors, which play pivotal roles in plant growth, development, and stress response [1]. In switchgrass, 42 *TCP* genes were identified from the genome and they were unevenly distributed on the chromosomes. The number of *TCP* genes in switchgrass is approximately twice that in Arabidopsis and rice, which have 24 and 21 *TCP* members, respectively [5]. No tandem repeats occurred in the evolutionary process in switchgrass *TCP* genes. So, large enrichment of switchgrass *TCP* genes was presumably due to the allotetraploid event. Furthermore, Ka/Ks analysis between the *PvTCPs* and *OsTCPs* was carried out, and the results that showed approximately 2/3 orthologous *PvTCP* genes, compared to *OsTCP* genes, are selected by natural selection pressure (Table 2), which might be due to the divergency between rice and switchgrass, at least 50 Mya [31]. As reported previously in *PvC3H* genes, the two sets of subgenomes of switchgrass originated from two closely diploid progenitors [31]. So, we speculated that *PvTCP* genes existed as paralogous gene pairs, which evolutionarily derived from the two sets of subgenomes, respectively. These results were also supported by previous studies in *PvSPL* genes and *PvARF* genes [32, 33].

The *TCP* gene family was classified into three clades, named as clade PCF, CYC/TB1, and CIN [5]. Similarly, PvTCP proteins were phylogenetically divided into those three clades in our study as well. Members that belonged to clade PCF have a four amino acid deletion in the basic helix-loop-helix (bHLH) conserved domain compared with clades CYC/TB1 and CIN (Figure 3). Exon/intron arrangement and motif location of *PvTCP* members were roughly conserved in the same clade but showed significant distinction among different clades (Figure 4). High similarity of the TCP members in switchgrass to other species, such as Arabidopsis and rice, suggested that *TCP* genes were highly conserved in plants, although there are great differences in gene numbers among different species [5]. Therefore, *PvTCP* genes would share similar functions with their orthologs in other species.

Previous reports about TCP roles mainly focused on cell cycle-mediated regulation of growth. TB1 is a major contributor to regulate apical dominance in maize [2]. PCF1 and PCF2 participate in DNA replication and repair, maintenance of chromatin structure, chromosome segregation, and cell-cycle progression by means of binding the promoter of the rice *PROLIFERATING CELL NUCLEAR ANTIGEN* (*PCNA*) gene, and CYC participates in the control of floral asymmetry in snapdragon [3, 4]. AtTCP4, a member in clade CIN, is critical in *Arabidopsis* floral organs [4]. Moreover, AtTCP4 can activate secondary cell wall biosynthesis and programmed cell death [22]. For those flower that predominantly expressed *PvTCP* genes in clade CIN, *PvTCP37*, *PvTCP13*/*40*, and *PvTCP14*/*16*, they may also take an important role in floral development, such as anther and pollen development. Not only the genes in clade CIN, but also the *TCP* genes belonged to clade CYC/TB1 can also control the floral asymmetry in *Lotus japonicus* (*LjCYC2* and *LjCYC3*) and *Pisum sativum* (*PsCYC2* and *PsCYC3*) [46, 47]. The expression levels of CYC/TB1 genes *PvTCP8*/*11* were relatively high in flower, which may affect the flower shape. Additionally, five PCF clade genes (*PvTCP1*/*4*, *PvTCP15*, *PvTCP27*, and *PvTCP39*) were predominantly high in flower and stem, which indicated that they might have a special function in floral development and cell wall biosynthesis. The expression profiles in different tissues of the *PvTCP* genes can help us study the detailed functions during switchgrass growth and development accurately in the future.

Several studies on the relationship between TCP proteins and plant abiotic stress have been reported [24, 25]. In *Agrostis stolonifera*, *miR319*-targeted *TCP* genes can respond to salt and dehydration stress and *Osa-miR319* overexpression transgenic creeping bentgrass improves salt and drought resistance [3]. *AsTCP5* transcript increased after 0.5 h salinity stress and then decreased at 6 h treatment point [3]. *OsTCP19* in shoots was upregulated under salt and drought stress in rice, and overexpression of *OsTCP19* in *Arabidopsis* can improve the abiotic tolerance of the transgenic plants [2]. In our study, we firstly analyzed the *cis*-acting DNA elements of the *PvTCPs*' promoters. It is revealed that a lot of photosynthesis, plant hormone signaling, and organ development regulatory elements were accumulated, such as S000449, S000265, and S000454 (Table 3). In addition, about 40% of *cis*-acting DNA elements were related to biotic and abiotic stress response, especially to salt and drought stress, such as S000407, S000144, S000447, and S000174. Subsequently, the expression pattern of *PvTCPs* was tested in switchgrass seedling when exposed to 250 mM NaCl. As described here, 29 out of 42 *PvTCPs* showed a trend of regulation under salt treatment but seemed to follow the different response patterns. *PvTCP17*/*18*, the homologous gene of *OsTCP19* in switchgrass, also can be induced under salinity condition, and their expression levels were nearly 2.3-fold higher than the control. Besides, about 69% of *PvTCPs* were response to salt stress, but the regulatory mechanism was not elucidated. Our study would provide great assistance for establishing the regulatory network about salt tolerance based on the transcription level in switchgrass.

## 5. Conclusion

In this study, we conducted a genome-wide analysis for the switchgrass *TCP* gene family to reveal their genome organization, phylogeny, gene structure, motif localization, function prediction, and expression profiles in different tissues and when exposed to salt treatment. A total of 42 TCP proteins were identified and phylogenetically divided into three clades; 29 of the *PvTCP* genes respond to salt treatment. It will provide us not only an insight of prediction and selection for *TCP* gene functions but also an information to exploit much more important gene resource for creating new germplasm in the future.

## Figures and Tables

**Table 1:** Overview of *TCP* genes in switchgrass.

Gene name^a^	Gene ID^b^	ORF length (bp)	Deduced polypeptide			Chr	Chr location
			Length (aa)	MW (kDa)	pI		
*PvTCP1*	*Pavir.1KG397100*	663	220	22.53	9.83	01K	63645349-63646113
*PvTCP2*	*Pavir.1KG510200*	1188	395	39.93	9.42	01K	75765762-75768115
*PvTCP3*	*Pavir.1KG552700*	681	226	23.00	9.79	01K	79521775-79523415
*PvTCP4*	*Pavir.1NG030900*	426	141	14.49	10.42	01N	3887213-3889118
*PvTCP5*	*Pavir.1NG539700*	1206	401	40.27	9.42	01N	89471749-89473804
*PvTCP6*	*Pavir.1NG547900*	663	220	22.50	10.09	01N	96987327-96989924
*PvTCP7*	*Pavir.2KG036700*	957	318	33.85	6.29	02K	5031244-5036367
*PvTCP8*	*Pavir.2KG296300*	801	266	28.62	6.05	02K	65347281-65349763
*PvTCP9*	*Pavir.2NG040500*	1293	430	45.36	9.32	02N	5718258-5719550
*PvTCP10*	*Pavir.2NG168500*	933	310	32.42	10.10	02N	32012380-32013416
*PvTCP11*	*Pavir.2NG320400*	795	264	28.39	6.21	02N	59626967-59628392
*PvTCP12*	*Pavir.2NG441900*	990	329	33.63	4.95	02N	81045305-81046294
*PvTCP13*	*Pavir.3KG357500*	870	289	30.21	8.93	03K	28804800-28807508
*PvTCP14*	*Pavir.3KG547300*	870	289	30.66	6.38	03K	69673046-69678099
*PvTCP15*	*Pavir.3NG031100*	1200	399	40.43	5.99	03N	2368139-2370112
*PvTCP16*	*Pavir.3NG279000*	864	287	30.06	5.96	03N	52604278-52608164
*PvTCP17*	*Pavir.4KG172900*	1197	398	40.38	8.97	04K	10928170-10929640
*PvTCP18*	*Pavir.4NG098900*	1173	389	39.50	7.83	04N	13829961-13832629
*PvTCP19*	*Pavir.4NG231900*	978	325	34.15	6.29	04N	19989691-19991909
*PvTCP20*	*Pavir.5KG544700*	1251	416	44.33	6.38	05K	94391370-94392775
*PvTCP21*	*Pavir.5KG556600*	837	278	29.62	8.08	05K	95365227-95369286
*PvTCP22*	*Pavir.5KG742600*	978	325	33.98	6.37	05K	113279411-113281724
*PvTCP23*	*Pavir.5NG501800*	1272	423	45.00	6.41	05N	86933569-86934999
*PvTCP24*	*Pavir.5NG508900*	531	176	18.87	9.75	05N	87491419-87493406
*PvTCP25*	*Pavir.6KG270000*	849	282	30.48	6.80	06K	55905938-55907215
*PvTCP26*	*Pavir.6KG395100*	1065	354	36.50	5.51	06K	70566109-70567758
*PvTCP27*	*Pavir.6NG051800*	1215	404	42.02	9.02	06N	10745904-10747230
*PvTCP28*	*Pavir.6NG140000*	711	236	25.40	5.61	06N	58122711-58123421
*PvTCP29*	*Pavir.6NG344700*	1329	442	45.95	8.82	06N	77613527-77615377
*PvTCP30*	*Pavir.7KG023900*	525	174	18.14	8.25	07K	23221010-23224633
*PvTCP31*	*Pavir.7KG255700*	606	201	20.79	10.01	07K	56383609-56384723
*PvTCP32*	*Pavir.7NG066100*	285	94	9.70	4.59	07N	15663289-15674390
*PvTCP33^ζ^*	*Pavir.7NG333200*	603	200	20.79	9.82	07N	55200631-55201233
*PvTCP34*	*Pavir.8KG079400*	1209	402	41.22	8.77	08K	9383778-9386025
*PvTCP35*	*Pavir.8NG062800*	1191	396	40.65	9.13	08N	8490834-8493093
*PvTCP36*	*Pavir.9KG031700*	1110	369	39.15	8.55	09K	2411064-2412737
*PvTCP37*	*Pavir.9NG079800*	1158	385	39.42	9.32	09N	5336448-5340260
*PvTCP38*	*Pavir.9NG142700*	1116	371	39.78	8.39	09N	13496559-13498279
*PvTCP39*	*Pavir.J125500*	582	193	19.25	10.19	scaffold14987	1395-1995
*PvTCP40*	*Pavir.J227000*	888	295	31.09	8.91	scaffold20	54419-56973
*PvTCP41*	*Pavir.J362100*	1335	444	46.34	6.78	scaffold276	1-2111
*PvTCP42*	*Pavir.J675700*	1353	450	46.78	6.67	scaffold7087	83-2442

^a^Gene name referred to the identified *PvTCP* genes in switchgrass in this study. ^b^Gene ID in Phytozome v12.0 database. *^ζ^*Corrected TCP genes by PCR and PviUTs database (https://switchgrassgenomics.noble.org/).

**Table 2:** Ka/Ks ratio of *TCP* orthologous genes between switchgrass and rice.

Orthologous genes	Ka/Ks ratio	Selection pattern
*PvTCP1*/*4* vs. *OsTCP7*	0.026	Purifying selection
*PvTCP39* vs. *OsTCP7*	99.000	Positive selection
*PvTCP31*/*33* vs. *OsTCP17*	99.000	Positive selection
*PvTCP15* vs. *OsTCP28*	99.000	Positive selection
*PvTCP27* vs. *OsPCF3*	99.000	Positive selection
*PvTCP34*/*35* vs. *OsPCF3*	99.000	Positive selection
*PvTCP30*/*32* vs. *OsPCF1*	99.000	Positive selection
*PvTCP2*/*5* vs. *OsTCP9*	99.000	Positive selection
*PvTCP17*/*18* vs. *OsTCP19*	1.250	Positive selection
*PvTCP19*/*22* vs. *OsTCP6*	26.467	Positive selection
*PvTCP12* vs. *OsTCP25*	99.000	Positive selection
*PvTCP26*/*29* vs. *OsPCF2*	0.552	Purifying selection
*PvTCP36*/*38* vs. *OsTB1*	0.847	Purifying selection
*PvTCP8*/*11* vs. *OsTCP24*	0.474	Purifying selection
*PvTCP25*/*28* vs. *OsTCP22*	0.516	Purifying selection
*PvTCP41*/*42* vs. *OsPCF5*	99.000	Positive selection
*PvTCP21*/*24* vs. *OsTCP5*	99.000	Positive selection
*PvTCP13*/*40* vs. *OsTCP18*	0.665	Purifying selection
*PvTCP14*/*16* vs. *OsPCF8*	99.000	Positive selection
*PvTCP37* vs. *OsPCF6*	0.032	Purifying selection
*PvTCP7*/*9* vs. *OsTCP21*	99.000	Positive selection

**Table 3:** Putative *cis*-acting DNA elements in the promoter of *PvTCP* genes.

Clade name	Element no.^a^	Element name^b^	Signal sequence^c^	Putative function^d^	FO^e^
PCF	S000449	CACTFTPPCA1	YACT	Photosynthesis	237
	S000265	DOFCOREZM	AAAG	Photosynthesis; leaf and shoot development	213
	S000454	ARR1AT	NGATT	Cytokinin response	161
	S000198	GT1CONSENSUS	GRWAAW	**HR reaction** ^**f**^ **; systemic acquired resistance**	146
	S000407	MYCCONSENSUSAT	CANNTG	**Abiotic stress; salinity stress**	143
	S000144	EBOXBNNAPA	CANNTG	**Salinity stress**; phenylpropanoid biosynthesis	143
	S000501	CGCGBOXAT	VCGCGB	Calmodulin; auxin response	108
	S000447	WRKY71OS	TGAC	**Biotic and abiotic stress**; GA response	97
	S000378	GTGANTG10	GTGA	Pollen development; pectin regulation	95
	S000493	CURECORECR	GTAC	**Copper; oxygen; hypoxic reaction**	92
	S000245	POLLEN1LELAT52	AGAAA	Pollen development	91
	S000415	ACGTATERD1	ACGT	Photosynthesis	74
	S000462	NODCON2GM	CTCTT	Root nodulin	66
	S000203	TATABOX5	TTATTT	Glutamine synthetase	45
	S000457	WBOXNTERF3	TGACY	**Jasmonic acid response**	43
	S000179	MYBPZM	CCWACC	Flavonoid biosynthesis; seed development	37
	S000176	MYBCORE	CNGTTR	**Abiotic stress; salinity**; flavonoid biosynthesis	35

CYC/TB1	S000449	CACTFTPPCA1	YACT	Photosynthesis	87
	S000265	DOFCOREZM	AAAG	Photosynthesis; leaf and shoot development	53
	S000407	MYCCONSENSUSAT	CANNTG	**Abiotic stress; salinity stress**	48
	S000144	EBOXBNNAPA	CANNTG	**Salinity stress**; phenylpropanoid biosynthesis	48
	S000198	GT1CONSENSUS	GRWAAW	**HR reaction; systemic acquired resistance**	38
	S000454	ARR1AT	NGATT	Cytokinin response	33
	S000378	GTGANTG10	GTGA	Pollen development; pectin regulation	32
	S000447	WRKY71OS	TGAC	**Biotic and abiotic stress**; GA response	21
	S000482	SORLIP1AT	GCCAC	phyA; phytochrome; light response	17
	S000203	TATABOX5	TTATTT	Glutamine synthetase	14
	S000030	CCAATBOX1	CCAAT	**Heat shock response**	13
	S000103	SEF4MOTIFGM7S	RTTTTTR	Seed globulin	10

CIN	S000449	CACTFTPPCA1	YACT	Photosynthesis	153
	S000454	ARR1AT	NGATT	Cytokinin response	132
	S000265	DOFCOREZM	AAAG	Photosynthesis; leaf and shoot dvelopment	103
	S000407	MYCCONSENSUSAT	CANNTG	**Abiotic stress; salinity stress**	77
	S000144	EBOXBNNAPA	CANNTG	**Salinity stress**; phenylpropanoid biosynthesis	77
	S000501	CGCGBOXAT	VCGCGB	Calmodulin; auxin response	69
	S000198	GT1CONSENSUS	GRWAAW	**HR reaction; systemic acquired resistance**	64
	S000447	WRKY71OS	TGAC	**Biotic and abiotic stress**; GA response	49
	S000493	CURECORECR	GTAC	**Copper; oxygen; hypoxic reaction**	48
	S000378	GTGANTG10	GTGA	Photosynthesis; leaf and shoot development	43
	S000203	TATABOX5	TTATTT	Glutamine synthetase	27
	S000103	SEF4MOTIFGM7S	RTTTTTR	Seed globulin	26
	S000245	POLLEN1LELAT52	AGAAA	Pollen development	21

^a-c^The ID number, name, and signal sequences of the element in the online PLACE program (https://sogo.dna.affrc.go.jp/). ^d^The putative function of each element predicted by the online PLACE program and references from NCBI. ^e^Frequency of occurrence in the promoters of *TCP* genes in each clade. ^f^Characters in bold represent those functions related to stress response.

## Data Availability

No data were used to support this study.

## Supplementary Materials

Supplementary 1 Figure S1: ten conserved motifs in PvTCP analyzed by the MEME search tool. The height of each box represents the specific amino acid conservation in each motif.

Supplementary 2 Table S1: primers used in this study.

Supplementary 3 Table S2: list of TCP members used for phylogenetic relationship analysis.

Supplementary 4 Table S3: microarray data of PvTCP genes.

Supplementary 5 Table S4: the detailed information of *cis*-acting DNA elements of PvTCPs.
