# Supplementary figures and images for: Genome-Wide Analysis of the TCP Gene Family in Switchgrass (Panicum virgatum L.)
Source: Int J Genomics. 2019 Apr 9;2019:8514928. doi: 10.1155/2019/8514928 (PMC6481156; doi:10.1155/2019/8514928)

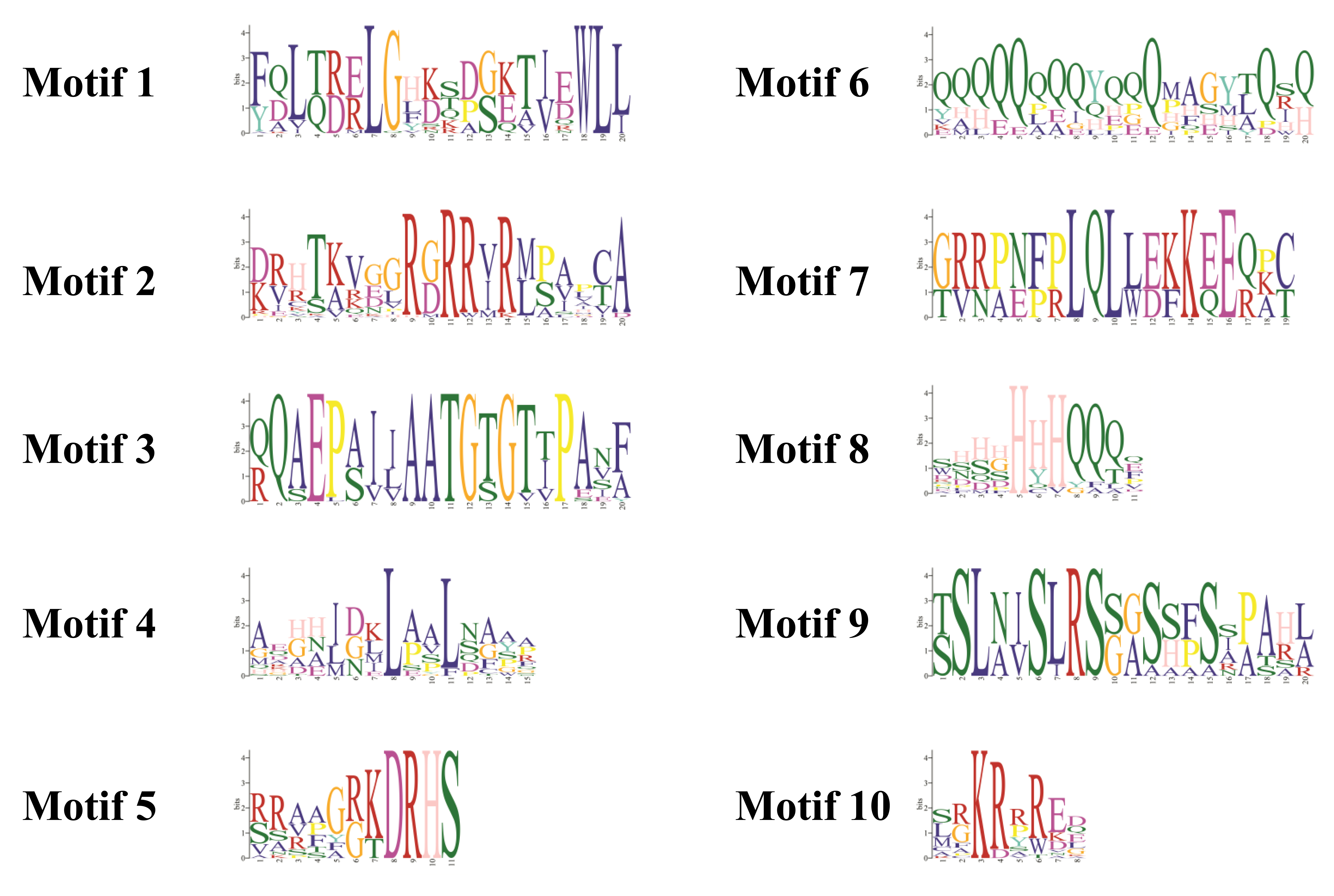

Supplement: Supplementary 1 — Figure S1: ten conserved motifs in PvTCP analyzed by the MEME search tool. The height of each box represents the specific amino acid conservation in each motif. [file 8514928.f1.jpg]
